# Interventions to Improve Clinical Coordination between Levels: Participatory Experience in a Public Healthcare Network in Xalapa, Mexico

**DOI:** 10.5334/ijic.5892

**Published:** 2021-11-01

**Authors:** Julieta López-Vázquez, Damián-Eduardo Pérez-Martínez, Ingrid Vargas, María-Luisa Vázquez

**Affiliations:** 1 Instituto de Salud Pública, Universidad Veracruzana, Av. Dr. Luis Castelazo Ayala s/n. Col. Industrial Ánimas, 91190 Xalapa, Veracruz, México; 2 Departamento de Pediatría, de Obstetricia y Ginecología, y de Medicina Preventiva, Universidad Autónoma de Barcelona, Bellaterra (Cerdanyola del Vallés) 08193 Barcelona, España; 3 Health Policy and Health Services Research Group, Health Policy Research Unit, Consortium for Health Care and Social Services of Catalonia, Avinguda Tibidabo 21, 08022 Barcelona, Spain

**Keywords:** care coordination, care integration, participatory action research, qualitative evaluation, health services research

## Abstract

**Introduction:**

Coordination of care can be improved through an intervention or a combination of several ones. In addition, it is recommended to encourage the active involvement of professionals in the design, implementation and assessment of coordination mechanisms.

**Objective:**

To analyse the factors that influence the implementation of participatively designed interventions and their effects on clinical coordination between levels of care in a public healthcare network of health services in Xalapa, Veracruz, Mexico.

**Methods:**

A qualitative, descriptive-interpretative study, for which individual interviews and discussion groups with a criterion sample of participants: Local Steering Committee and the Professional Platform. A content analysis, with mixed category generation and segmentation by intervention and topics, was carried out. According to the problem analysis, participants designed two sequential interventions: offline virtual consultation, and joint training meetings on maternal health and chronic diseases.

**Results:**

Respondents perceived a differentiated impact on clinical coordination according to intervention: greater in the case of joint maternal health trainings and limited for the chronic diseases meetings, as they were the offline virtual consultation was rarely used.

**Conclusion:**

The involvement of professionals in designing the interventions, as well as institutional support and reflexive methods for training, all decisively improved clinical coordination between levels.

## Purpose

The purpose of this manuscript is to share the learnings of the implementation process of two participatively designed interventions to improve coordination between levels of care, and to identify the key elements for the application of this type of strategies in Mexico and other contexts.

## Introduction

Coordination between levels of care represents a challenge for improving the effectiveness of health services in Latin America [1]. Recent studies have shown that in countries with fragmented health systems, such as México, there are poor information transfer, clinical disagreements and a limited accessibility between levels of care [2,3]. The causes of this include: organisational factors, such as insufficient time to carry out coordination activities [2,4] or the availability of coordination mechanisms [5,6] and interactional factors between professionals such as, lack of mutual knowledge and trust [2,4]. Although the policies aiming at strengthening primary care in Mexico [7,8,9] contemplate the improvement of coordination between levels of care, the few existing evaluations have shown a limited and difficult implementation of strategies and coordination mechanisms, attributed to the lack of context adaptation (i.e. insufficient technological resources or supplies to meet the recommendations provided by the Official Mexican Standards/Clinical Practice Guidelines) [10,11], or the use of more as an administrative requirement (for referring to another level of attention [referral and reply letter] or discharge [discharge report]), than for clinical coordination [12,13].

The limited adaptation of interventions to improve care coordination, which are designed and implemented vertically (top-down), into a local context is common in many health systems, not only in Mexico, and has been strongly criticised for being ineffective [14]. In contrast, participatory action research is characterized by cyclical, reflexive and flexible processes of planning, action and evaluation [15], and includes the participation of professionals in decision-making [16], this has proven to be effective to: a) motivate professionals to incorporate interventions into their healthcare practice [17], b) influence interactional factors that impact coordination between levels of care, such as mutual knowledge and trust [18,19] and c) design interventions adapted for the local context [16], which is decisive to favour their adoption and effectiveness [20,21]. Despite the potential benefits of participatory action research for the design and implementation of interventions, its application in health services is practically non-existent [19,22].

Care coordination is here defined as the harmonious connection of the different services needed to provide care to a patient throughout the continuum of care in order to achieve a common objective without conflict [23]. Three cross-level coordination types are considered: information coordination, clinical management coordination and administrative coordination; the first two are related to clinical aspects. The coordination of information refers to the transfer and use of clinical information from patients, while the coordination of clinical management involves the consistency of care, the accessibility and adequate patient follow-up [24]. One way in which care coordination can be improved is by conducting clinical management interventions at the micro level, with the introduction of a coordination mechanism or the combination of several. Those might be based either on standardising skills (continuous training), work processes (Clinical Practice Guidelines), or the outcome; or based on feedback between professionals (direct: telephone, e-mail), or through other mechanisms (inter-consultation, hospital discharge report or shared electronic medical record).

As in other areas of quality improvement, the active participation of professionals in the design, execution, and evaluation of strategies is recommended [25]. However, existing evaluations on the implementation of coordination mechanisms between levels of care using qualitative research approaches, are more common in high-income countries [26,27]. In Mexico, studies are limited to the use of mechanisms in one level of care [10,11,12,13,28,29] without considering the interaction between levels, despite its relevance for clinical coordination [30]. Clinical practice guidelines is the most-analysed mechanism, and the one for which barriers and facilitators to its implementation have been identified during implementation [10,11,28,29]. In contrast, referral and reply letters and hospital discharge reports are only evaluated as part of the clinical record (whether or not they are physically available, and without analysing the quality and relevance of the information) [12,13]. However, analysing the process to implement interventions, from the perspective of users (health professionals), plays a key role when identifying the results of care coordination, as well as in understanding the factors influencing their adoption, dissemination, and potential sustainability and replication [29,31].

The study is part of a broader investigation [30] (Equity-LA II; *www.equity-la.eu/en/*), which combines quantitative methods to analyse the effectiveness of interventions in improving care coordination [32] and continuity [33], with qualitative methods to evaluate the design and implementation process and the perceived contributions of interventions to improving coordination between levels of care from the perspective of the participants [34]. This article focuses on the experience in Mexico, delving into the similarities and differences between interventions, and complements previous local publications on care coordination [3,6,35] and continuity [36,37]. The objective of this article is to analyse the factors that influenced the implementation of participatively designed interventions and their effects on clinical coordination between levels of care in a public network of health services in Xalapa, Veracruz, Mexico.

## Methods

### Study area

The study was is located in Veracruz, one of the most populated states in the country, with more than eight million inhabitants [38] and a prevalence of chronic diseases and maternal mortality that exceeds the national average [39]. The study healthcare network is located in the capital of the state of Veracruz, Xalapa. This municipality is one of the most populated in the state, with 480,841 inhabitants, from which 59.46% lacks social security [38]. This population is cared for in the State Health Services network, object of this study. This healthcare network was chosen as it provides a continuum of health services, including primary care (seven units) and secondary care (two hospitals) services, for a defined population living in an urban area with a predominantly low or medium-low socioeconomic level and that was willing to participate [30].

### Intervention design and implementation process

The participatory process began when the Local Steering Committee was established. The Local Steering Committee led the design and implementation process of interventions and was integrated by state health managers and middle managers, with the research team acting as the facilitator. The Local Steering Committee the results of the baseline study on care coordination and then held 11 meetings with primary care professionals and four with secondary care professionals (totalling 225 participants), in order to present those results within the healthcare network. From these meetings, a group of volunteer professionals from both healthcare levels, involved in healthcare practice and interested in action, was consolidated. This group, called the Professional Platform, included 31 professionals from both levels (mostly physicians), whose role was to delve into the problems identified, select one and select the intervention to address it, as well as design and implement it, together with the Local Steering Committee).

The intervention design and implementation process were developed during two participatory action research cycles. During the first cycle, the Professional Platform and Local Steering Committee designed the offline virtual consultation between primary and secondary care physicians, the description and implementation of which are shown in ***Table 1***. When the intervention began to be implemented, restructuring in the State Ministry of Health caused changes in the Local Steering Committee (which was reduced from eight to six members, from which five members were new, no changes in the research team). The Professional Platform was also reduced (from 31 to 17). The offline virtual consultation would allow the identification of training needs, but due to the low use of the system, a survey was carried out to detect participating physicians training needs, their willingness to participate in joint training activities and other topics of interest. Maternal health and chronic diseases were among the most requested topics and 94% of primary care physicians and 100% of secondary care physicians (participating as training facilitators) expressed their willingness to participate. At the end of the first cycle, the Local Steering Committee membership was changed by the incoming health authorities (two members remained, while seven were incorporated, and one member of the research team was also replaced).

**Table 1:** Description of the interventions and their implementation process.


OFFLINE VIRTUAL CONSULTATION BETWEEN LEVELS		

	1ST CYCLE	2ND CYCLE

**Content**	Asynchronous virtual consultations in chronic diseases care via digital platform and protocol repository between primary care and secondary care physicians	Maternal and perinatal health is incorporated as a an area for consultation

**Trained physicians**	68 primary care physicians	2 secondary care physicians

	13 secondary care physicians	

**Number of consultations conducted**	6 consultations; accessed 43 times to look up information	5 consultations; accessed 165 times to look up information

**Duration**	6 months (October 2016 – April 2017)	8 months (May – December 2017)

**JOINT TRAINING MEETINGS**		

**Content**		Joint training meetings, based on clinical cases, on maternal and perinatal care and chronic diseases

**Number of sessions caried out**		4 maternal and perinatal health

		1 chronic diseases

		Maternal and perinatal health: 52 primary care 12 Gynecologists, 1 Clinical nutritionist

**Participants**		Chronic diseases

		20 primary care

		2 Internists

		1 Clinical psychologist, 1 Pneumologist, 1 Ophthalmologist, 1 Emergency physician, 1 Integralist physician

**Duration of implementation**		8 months (May – December 2017)


Based on the joint analysis (between the new Local Steering Committee and Professional Platform) of the results of the first cycle, and the review of the training needs identified, the Local Steering Committee decided to maintain and expand the offline virtual consultations and introduced the joint training meetings between levels (***Table 1***), starting the second cycle. These meetings, which were jointly planned for 12 sessions between the Local Steering Committee and facilitators, consisted of three-day seminars (eight hours/day) outside of the state health services, with the involvement of primary care physicians and facilitated by specialists in the network.

The joint training meetings were based on the joint analysis of clinical cases from the health care, through the application of reflexive training methods. These methods allow the analysis/reflection of one’s own practice to improve it, so they require the active participation of the clinician and facilitator [40]. This type of method stimulates communication, feedback, and clinical agreement, which contributes to increasing the resolution capacity of first care level physicians and improves the interaction and coordination between primary and secondary care [14].

### Study design

A qualitative descriptive-interpretive study was carried out to analyse the factors that influence the implementation of the participatively designed interventions and their effects on clinical coordination between levels of care, from the perspective of the participants.

### Analysis framework

The evaluation framework includes: the analysis of the effectiveness and perceived impact in relation to the coordination and continuity between levels of care (final and intermediate result) and, the analysis of the implementation process of the interventions (process results).

The evaluation framework used in this study is based on the three dimensions identified by Pettigrew and Whipp [41,42]: the context of the intervention -health system and policy (outer setting) and health services networks (inner setting)-, the content or characteristics of the intervention and the process of implementation. The last two are grouped into a single dimension in the participatory action research process

Over time, the context and process of implementation elements interact in a complex way, influencing the results of the intervention implementation process (process outcomes) and the intermediate and final results on coordination and continuity, between levels of care (health services outcomes). The focus was the results as from the point of view of the participants

### Sample

A sample was designed by criterion of key actors who had participated in any of the different stages of the process of selection, design and implementation of the interventions, in order to have all the possible discursive variants (members of the Local Steering Committee and members of the Professional Platform), with maximum variation according to their role in the intervention. The sample consisted of 15 informants: nine members of the Local Steering Committee (four managers and five middle managers) and six of the Professional Platform (three primary care and three secondary care physicians) who were interviewed individually, in a discussion group or in both (***Table 2***).

**Table 2:** Final composition of the sample of informants.


		STATE MANAGERS	MIDDLE-RANKING OFFICIALS	SECONDARY CARE PHYSICIANS	PRIMARY CARE PHYSICIANS

**Individual Interview|**	**Local Steering Committee**	2 (Male)/ 1 (Female)	–	–	–

	**Professional Platform**	–	–	–	1 (Male)

**Discussion Group 1**	**Local Steering Committee**	–	2 (Male)/1 (Female)	–	–

**Discussion Group 2**	**Local Steering Committee**	–	1 (Male)/1 (Female)	–	–

	**Professional Platform**	–	–	3 (Male)	2 (Female)

Discussion Group 3*	**Local Steering Committee**	1 (Male)	3 (Male)	–	–

	**Professional Platform**	–	–	–	1 (Male)/1 (Female)


* Discussion Group conducted in 2 sessions.

### Data collection

Data collection was carried out through discussion groups and individual semi-structured interviews, to delve into some issues. Topic guides were developed for each cycle and intervention, the guides included: opinions on the design and implementation process; influencing factors on the implementation of interventions (content of the interventions, process, and context related to the health system and its policies, healthcare or professionals); and perceived results on care coordination and influencing factors. According to the established criteria, informants were identified and then asked to join by considering their attendance at meetings held by the Local Steering Committee and Professional Platform as well as at joint training meetings. There were no refusals to participate. The discussion groups were carried out outside their institution (90 min on average), while individual interviews were conducted at the workplace of the informants (45 min average duration) and were literaly recorded and transcribed. The fieldwork took place between March and July of 2018.

### Data analysis and quality of information

Thematic analysis was carried out using ATLAS.ti® software. Data were segmented by intervention and topics. Analysis categories were generated in a mixed way (from the topic guide and emerging from the discourses). Topics were identified, coded, re-coded and classified, in order to find out similarities and differences among informants. To ensure good quality of results, the information was triangulated using several techniques and various analysts who had a good understanding of the context were involved. The preliminary results were presented, and feedback was given over two meetings (with seven informants), allowing us to confirm the findings by taking into account the participants opinions on the analysis.

### Ethical considerations

A confidentiality agreement was signed with the institution. Involvement of each informant was free and voluntary expressed in written form by signing a an informed consent. The recordings and transcriptions were coded to guarantee the anonymity of informants.

## Results

A differentiated impact of each type of intervention on care coordination and related factors, as well as differences in the factors influencing the implementation process, emerged from in the informants discourses. Differences were observed according to the informant profile.

### Offline virtual consultations

**Figure 1:**
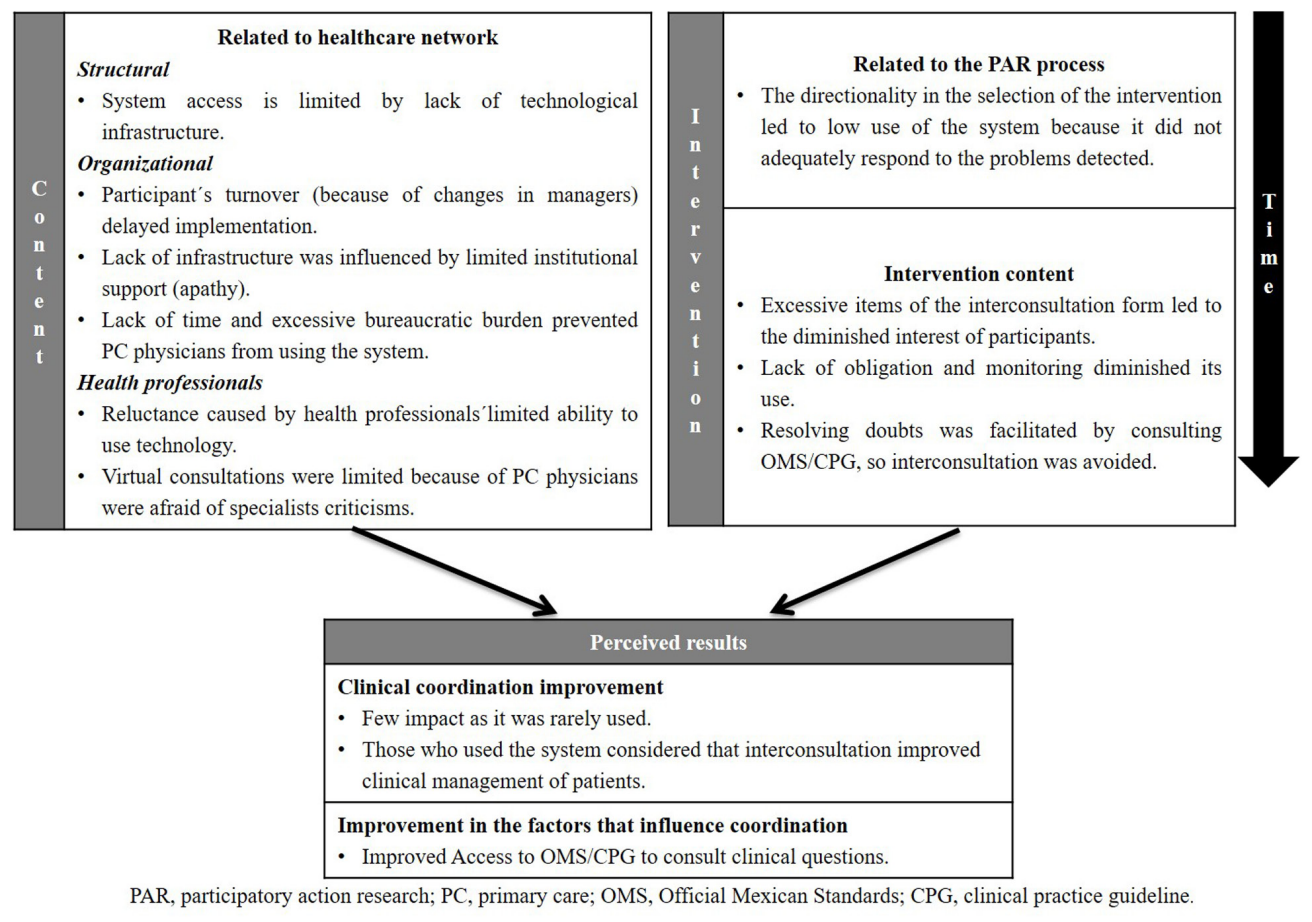
Offline virtual consultations: results perceived on care coordination and related factors.

Limited results were obtained in terms of improved coordination or influencing factors. Several unfavourable contextual factors related to the healthcare network were identified, which, in conjunction with the weaknesses shown in the participatory process regarding design and content of the offline virtual consultations, explain why it was rarely used (***Figure 1***).

#### Results on inter-level coordination and related factors

The limited impact of the offline virtual consultations on coordination between levels of care and related factors, which was attributed to its limited use, emerged in the informants’ discourse:

“I am terribly sorry, because only two questions were asked during the whole process, no more questions…no changes regarding referrals, not at all, they don’t use the system, two questions, after many months, actually it’s critical.” (Professional Platform/Primary Care)

However, a professional platform/primary care physician considered that their clinical management of patients had improved due to the specialist’s response (***Table 3a***), while some Professional Platform informants pointed out its impact on using other coordination mechanisms between levels of care, such as the Official Mexican Standards/Clinical Practice Guideline (***Table 3b***).

**Table 3:** Examples of textual quotations regarding opinions on the results of the offline virtual consultations and factors influencing their implementation.


**Results on improved coordination of clinical management**	
a- Interconsultation improves clinical management of patients	“The interconsultation helped me exchange management concepts with the specialist, and. obviously, his responses about the patient to keep him under control and happy.” (Professional Platform/Primary Care)

**Factors that influenced on offline virtual consultations implementation** Contextual	
b- The system improves access to Official Mexican Standards Clinical Practice Guideline	“If I had any doubts, then I checked the Official Mexican Standards Clinical Practice Guideline to get answers … then I practically accessed to get information … [which was] very useful for me” (Professional Platform/Primary Care). “Moreover, in the system we could also make enquiries about pregnancy, childbirth and puerperium, since the official standards were there, no many problems. Regarding chrome diseases, we began to focus on manuals and the Clinical Practice Guideline.” (Professional Platform/Primary Care)

**Related to the healthcare network** Structural	
c- System access is limited due to a lack of infrastructure	“First, we didn’t have internet in fact we just got internet a few months ago, and because there was none… at the health centre you couldn’t ask questions. If I consulted information from the system I had to connect fron home” (Professional Platform/Primary Care). “The internet system implied connectivity in the units, which they have, but that connectivity is not very efficient, computers are necessary, not all of the units had computers… and many units with computers did not have connectivity. That connectivity was even paid by physicians.” (Local Steering Committee/Manager)
Organisational	
d- Providing technological infrastructure is influenced bymanager’s limited support or interest	“When we spoke with the authorities, some situations that could hinder the system (the offline virtual consultations) were perceived. One of them was the lack of computers and internet at the Health Centres, for which a census was carried out… and after the census, the Ministry of Health committed to correct this situation… when a government institution is committed, there is 20% of certainty they will comply, while 80% they won’t comply. That happened here or at least primary care physicians still complain about this and they still report that they do not have a convenient internet access and they sometimes do not even have computers.” (Local Steering Committee/Manager). “After all the paperwork one has to fill out (in primary care), in the end you are going to forget about the system (offline virtual consultationes), it was not intentional. However, somehow SESVER got involved and focused much more on obstetrics, so the training in obstetrics was very good. If SESVER would engage with the system (offline virtual consusltations) or with chronic patients, or with comprehensive care… not only with obstretics, I think it would really be much easier for us… I still feel that the main cause is that SESVER did noy join this, as is not part of their work plans.” (Professional Platform/Primary Care).

**Related to professionals**	
e- Fear of criticism diminishes interconsultations	“I think the problem (that the system would not work) is they are afraid of being criticised, they will criticise us… criticism is not going to favour absolutely anything, the idea was to unite in order to establish a more open and cordial dialogue, avoiding precisely that feeling that we are annoying.” (Professional Platform/Secondary Care)

**Related to participatory action research process and intervention content**	
f- Directionality when selecting causes for the system to be rarely- used, as problems identified are not adequately responded	“The research team had already visualised that they wanted to implement this system…surely they had already planned to do so…this is an important issue”. (Professional Platform/Primary Care) “It seemed to me that this search (for interventions to be implemented) by some members of the university team was biased…with the causes that were identified in those studies, then the action plan began to be designed …we were taking risks to start designing solution strategies that might not solve the problem and at the end of the period we were going to realise it did not work, but hey, it is part of the investigation, if something is set in motion and does not work, well, at least it is proven.” (Professional Platform/Primary Care)
g- Too many items in the interconsultation form diminishes interest of participants	“At first I was involved, I tried to type info of patients, do the summaries and all that, but the truth is that I could devote that time to make my notes, organise my files, I did it for a while, when I realised that it really took away a lot of my time.” (Professional Platform/Primary Care) “The items were practical for me (interconsultation format), those considered as necessary to make consultations during the meeting held by primary, secondary and tertiary physicians were included.” (Local Steering Committee/Manager)


#### Factors influencing its implementation

Informants identified contextual factors related to the healthcare network, as well as the content of the intervention that influenced implementation of the offline virtual consultations.

**Healthcare network**. Several factors that limited the use of the offline virtual consultations emerged from the discourse of both groups of informants: 1) Structural factors, such as the lack of computers and intermittent internet connection, mainly for primary care use, and limited or prevented access at the workplace (***Table 3c***).

**Organisational factors**. According to Professional Platform informants, changes in the Local Steering Committee and Professional Platform (due to changing of managers) delayed the start of implementation:

“Staff turnover in both levels of care, change of officials, training people all the time to continue the project, well, yes, it was going to affect the project (was delayed), but then this occurs at the institutional level.” (Professional Platform/Primary Care)

Moreover, Local Steering Committee informants identified a lack of institutional support, with the new managers showing little interest during the first months of implementation, as they did not provide computers and internet in some primary care units during the first cycle; thus, the offline virtual consultation were not used (***Table 3d***).

In addition, informants identified that there was no time available for primary care physicians to use the system due to excessive bureaucratic burden:

“Primary care (…) they said that the form required a lot of time. It’s just that they have to fill out too many forms, so they are filling out all the forms while treating patients. So I think this is the reason why the system was not used correctly.” (Local Steering Committee/Manager)

**Professionals**. The limited ability of some physicians to use computers manifested as resistance toward using them:

“(The physicians) are reluctant to use the computers, that is, I don’t even know how to turn it on, I don’t know how to use it, I’m going to break it and the staff has been trained over and over again.” (Local Steering Committee/Manager)

Moreover, primary care physicians were afraid of criticism from specialists. Both factors contributed to a lack of interconsultations being generated (***Table 3e***):

“We complained about the dialogue with the secondary care, which was not adequate to provide integrated care service as physicians…maybe also, we are afraid to ask questions to specialists as we can be ridiculed.” (Professional Platform/Primary Care)

**Participatory action research process and intervention content**. Although most informants considered that the choice of the offline virtual consultations had been by consensus, several Professional Platform members stated that it was rarely used, due ti the directionality when the research team chose this intervention, resulting in an intervention that did not address properly the problems detected (***Table 3f***).

“The institutional decision-makers, researchers and people working for the primary and secondary services of the network…saw several lines of action, linking the primary and secondary care using some electronic mechanism the one that was seriously considered.” (Local Steering Committee/Manager).

On the other hand, the Professional Platform considered, unlike the Local Steering Committee, that the information required by the form to carry out the offline virtual consultations was excessive and required a long time to complete. Thus, interest in carrying out interconsultations was diminished (***Table 3g***). One member of the Professional Platform stated that the lack of obligation and supervision also contributed to the system rarely being used:

“Unfortunately, if people are not spurred, then they don’t work, but if you implement, a well-implemented program, but above all a demanding one…well look, we have a system for you to communicate with the internist, you do it, because I am demanding that you do it for me, but if I am indulgent with you, things won’t move on, this is what I detected.” (Professional Platform/Primary Care)

Finally, several informants of the Professional Platform group stated that checking information on Official Mexican Standards/Clinical Practice Guideline consultation via the system resulted in the resolution of doubts, making the issuance of an offline virtual consultations unnecessary:

“Practically if I had any doubts, when I checked the clinical practice Guideline or the standards my doubts were solved…then I practically accessed to check the information…very, very useful for me.” (Professional Platform/Primary Care)

### Joint training meetings

**Figure 2:**
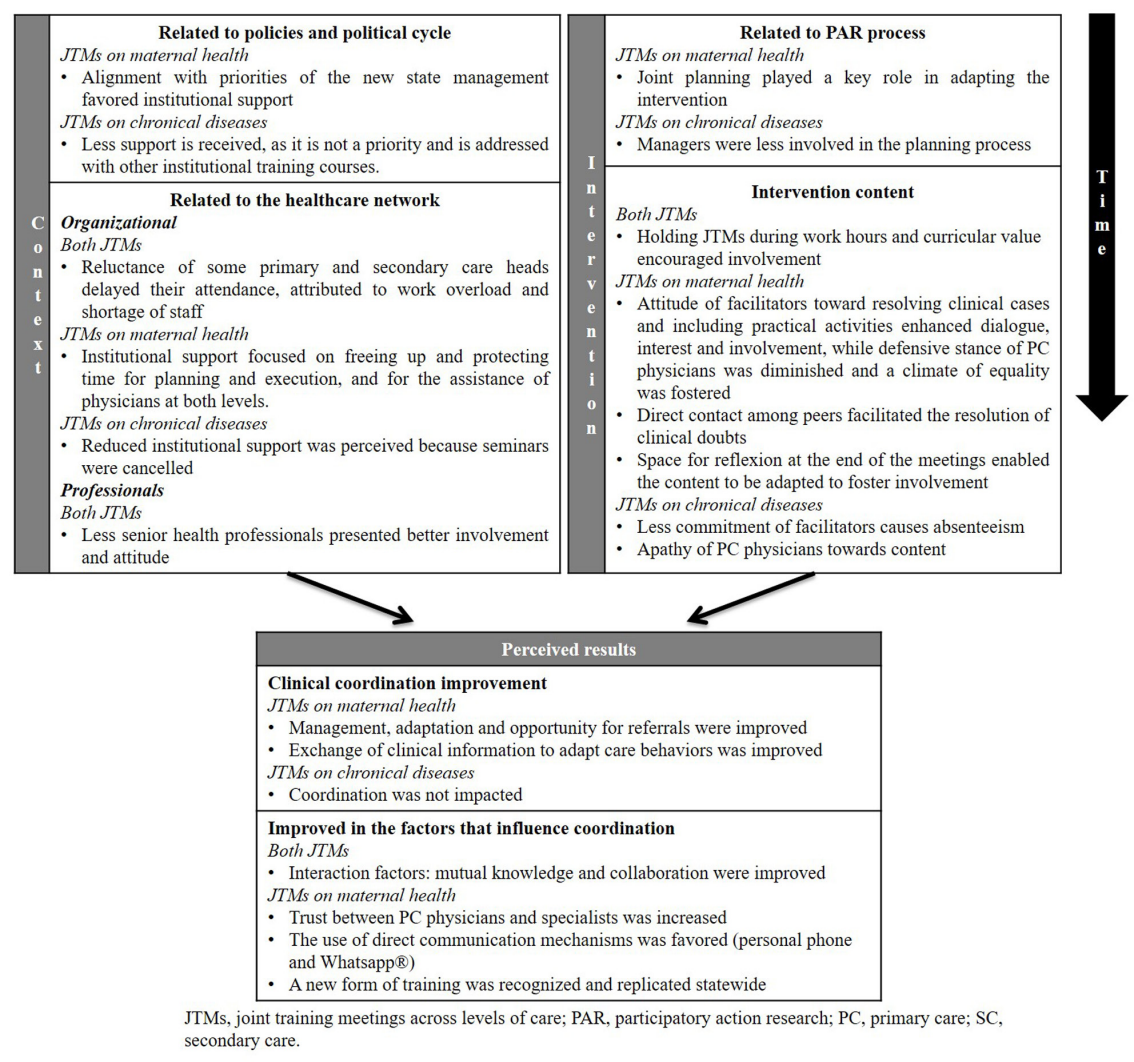
Joint training meetings: results perceived on care coordination and influencing factors.

Had joint training meetings on maternal health had an impact on the coordination of care and its influencing factors. Several factors favourable for the development of this coordination were identified; a change in authorities to ones with new priorities led to institutional support for the intervention, in conjunction with the participatory nature of its design, and the use of a reflexive method was implemented. On the other hand, regarding the joint training meetings on chronic diseases, a low impact was observed due to its limited implementation, which was related to the lower institutional support; thus, neither coordination nor its factors were affected (***Figure 2***).

#### Results on coordination between levels of care and related factors

The contribution of joint training meetings on maternal health to the coordination of clinical management and clinical information between levels and to the interactional factors between primary care and secondary care physicians, in contrast from joint training meetings on chronic diseases, was emerged from the discourse of informants.

##### Clinical coordination

According to informants, the joint training meetings on maternal health improved clinical management of patients, with a better adaptation and timeliness of their referral to secondary care and of the exchange of information between levels (***Table 4a***). There was also an improvement in the exchange of clinical information via direct communication, in order to adapt obstetric care behaviours (via personal telephone and WhatsApp):

“We began to notice after the seminar…that the primary care physician consulted more the network leader (consultant), consulted more and asked questions related to referrals, came closer to the hospital, but it was not just the fact of making referrals and that’s it! No! Now it is to make referrals so that they aren’t rejected” (Local Steering Committee/Manager).

**Table 4:** Examples of verbatin regarding opinions on results on coordination of joint training meetings and factors that influenced their implementation.


**Results on improved coordination of clinical management**	
a- Treatment, adequacy and relevance of referrals were improved	“Something very important is that (in a referral) the woman was there with both the proper documentation and the clamp-crush technique well done Then they had already learned this in the seminar, so, how have you noticed this? Changes may be very subtle, but can be noticed” (Local Steering Committee/Manager). “There was an impact on matters regarding delicate patients, who have arrived at the hospitals in better conditions and they were previously treated, that means that there was a positive result in terms of contact between the secondary, tertiary and primary levels of care.” (Local Steering Committee/Secondary Care)

**Results on improved factors related to coordination**	
b- Use of direct communication	“Today everyone has a phone with WhatsApp… they take a photo and send me the referral and I’ll send you the reply letter back and I’ll tell you this, send that send, give me that very quickly.’’ (Local Steering Committee/Manager)

**Factors that influenced on holding joint training meetings** Contextual related to policies and political cycle	
c- Alignment with policies favoured institutional support when considered as an opportunity	“When it turns to maternal death, the first seminar was in large part due to the support received by the Directorate of Medical Care – we have to abate this problem that is screwing us up, and, suddenly, the problem became bigger, and, well, we have to see how to resolve.” (Local Steering Committee/Manager)

**Related to participatory action research process and intervention content**	
d- Attitude of facilitators, clinical case resolutions and practical activities enhanced involvement and fostered a climate of equality	“Even during the same seminar, some of the secondary care physicians were really conflicted with the relationship between primary and secondary care, but in the last seminars no longer. Even I think that the word spread about – those who attend (specialists.) They are not going to ask questions or be rude – but in the last seminar one or two took out their frustrations… but most of them attended with an open perspective to leans and improve, that’s what interaction is about” (Local Steering Committee/Secondary Care). “Training dynamics shown by secondary care physicians was important, as they were perceived as equals’ And that was the difference from other trainings.” (Local Steering Committee/Manager)
e- Topics (maternal health) and curricular value encouraged involvement	“Everything related to maternal and child health is much more frequent, it is a priority, I am not saying that chronic degenerative diseases are not important in fact, they are one of the main causes of morbidity and mortality… the staff have a certain affinity with health problems, it seems that when chronic degenerative diseases see the problem with the user is not going to be solved immediately, our mentality seems to change” (Professional Platform/Primary Care). “It was fortunate that maternal health is now considered, because it is a daily walk, perhaps we don’t receive many chronic degenerative patients, but pregnant women do, every day.” (Professional Platform/Primary Care) “(The incentive) for the primary care physicians was to give them their duly registered certifications, so that they can use them for job promotion and so on.” (Local Steering Committee/Manager)

**Related to the healthcare network (organisational and individuals)**	
f- Freeing up and protecting time of participants facilitated design and implementation	“In order for me to get fully involved, I only had to say I was approved (by the Directorate of Medical Care), so I can enjoy a certain autonomy of the staff under my command, use the resources I was allocated for activities for both (the joint naming meetings) and the strategic plan” (Local Steering Committee/Manager). “It was our turn to establish the leadership in terms of training, managing with the health jurisdiction the call for the groups of participants, discuss with the directors and managers of the regional hospitals the participation of the teams of facilitators and see how to move forward (because of resistance).” (Local Steering Committee/Manager)

g- Resistance from some primary and secondary care beads delayed involvement of physicians	“There was a certain resistance (from management positions), obviously, to allow the primary care physicians to be out for three days, and for the secondary care… no way you are going to take away my specialist that gets the job done, because he has to deliver a gaining course.” (Local Steering Committee/Manager)

h- Less support resulted in cancelling seminars on chronic diseases	“That political support for other programs, which perhaps does not occur for COPD or chronic degenerative diseases, because they always have programs for diabetes, but they aren’t a priority… (The second replication of the course on chronic diseases) was cancelled in December.” (Professional Platform/Secondary Care)

i- Less seniority encourages interest to get involved	“In the last courses we saw more involvement, more acceptance by physicians, which has a lot to do with staff s seniority, staff that is very old have many tricks and, indeed, are very resistant to methodology.” (Local Steering Committee/Manager)


##### Factors influencing coordination

The informants perceived that joint training meetings had an impact on interactional factors, since personal knowledge between physicians was allowed; thus, collaboration was favoured:

“This close relationship among people working here is fostered, because we no longer perceive them so distant…we perceive them as members of the same team…attitude towards the patient changes when you know that he is received by someone you know” (Professional Platform/Primary Care).

However, only joint training meetings pertaining to maternal health increased trust between primary care and secondary care physicians:

“It is important that contact between specialists and primary care physicians continues to exist, since trust between both levels is built, closer ties are created and the path to treat patients is better managed, so a better conversation without fear of teasing can get started… that shouldn’t get lost” (Local Steering Committee/Secondary Care).

And favoured the use of direct communication (personal phone number and WhatsApp) (***Table 4b***):

“They call me directly, they send me a message and then they say, ‘Hey, I’m…from this health centre, there is a female patient with me that…so do I send it just like this? What do I do?’ There is a very close contact” (Local Steering Committee/Secondary Care).

Additionally, informants stated that joint training meetings were a new form of institutional training to facilitate agreement on clinical management between physicians from both levels of care:

“Under this structure we have designed courses, obviously with other contents a little more practical…workshops that are more experiential than the simple case review, but the same for all general practitioners, gynaecologists, surgeons who involved in gynaecology, and anaesthesiologists” (Local Steering Committee/Manager).

And this practice was subsequently replicated in the rest of the state:

“Since the research was planned for 10 and reached 20, then it is even more satisfactory. It reached 20 because, as you say, something that was intended to be done in, in a few health centres of the network, it turns out that it was extended state wide, to all centres located in the state, and to all primary and secondary physicians from the state of Veracruz” (Local Steering Committee/Manager).

#### Factors influencing implementation of joint training meetings

Differences were found in the factors influencing joint training meetings implementation according to the type of informants; the Local Steering Committee focused on those related to the political context, while the Professional Platform emphasised those related to the intervention content. To a lesser extent, other factors related to the healthcare network also emerged.

**Policies and political cycle**. Informants noted that the new health authorities prioritised maternal health over chronic diseases, as this was were aligned with the Maternal Health State Plan, which increased institutional support for maternal health seminars (***Table 4c***):

“The Directorate of Medical Care is in charge of addressing the strategic plan for maternal and perinatal health in the state to reduce maternal death and infant death…so who is going to help me? The project, of course” (Local Steering Committee/Manager).

In this sense, the informants perceived less support in the joint training meetings on chronic diseases, as they were not a priority for the state:

“And it is also very evident that the change was more noticeable for pregnancy, childbirth and puerperium care than for the chronic conditions, perhaps that is why, because of the same interest, as they had already set their sight on this, then they are working flat out and it already occurs for chronic conditions, but to a lesser extent” (Professional Platform/Primary Care).

Moreover, they were addressed by other institutional trainings:

“At least in the health centre we try to update our knowledge, we are continuously attending refresher courses, especially for chronic diseases.” (Professional Platform/Primary Care)

**Participatory action research process and intervention content**. In the Local Steering Committee’s discourses, that the joint planning of joint training meetings on maternal health by both the Local Steering Committee and the facilitators (specialists) played a key role in adapting them to the training needs stated by physicians from both levels and getting managerial support and interest of participants:

“I believe it is ideal, that is, everybody should get involved, participants, stakeholders, because I observed before filling this position that in many times the vertical interventions are not going to flourish, because they were probably conceived by a single person, but if we consider the needs of all parties and, obviously, also the solutions that each party can provide…it’s like making a tailored suit. Thus, I think that the opinion, the involvement of everybody in the different positions and fields of action, because obviously they made a tailored suit for needs of primary and secondary physicians, as well as the same area, our medical care area and the medical teaching department” (Local Steering Committee/Manager).

In contrast, for chronic diseases, the involvement of state managers who were members of the Local Steering Committee was lowered during planning:

“They said: ok, let’s get started by (planning) the seminars on chronic conditions…there, there has been a lack of communication between the directorates, for example, we could say Public Health, the Directorate of Medical Care… and the Sub-directorate of Education” (Local Steering Committee/Manager).

Regarding the training method, informants considered that the open attitude and absence of hierarchies shown by facilitators of the joint training meetings on maternal health, as well as including practical activities and resolution of clinical cases, enhanced dialogue, interest and involvement in the sessions. Thus, the initial defensiveness of primary care physicians was eliminated, a climate of equality (***Table 4d***) was fostered, and the resolution of doubts via direct communication between peers was promoted:

“Now, I was able to stay in touch with a gynaecologist, and any particular doubt I could not solve by reading (regarding CPG), so I could trust them, I was able to detect a greater close relationship” (Professional Platform/Primary Care).

On the other hand, informants agreed that, during the meetings on chronic diseases, some facilitators showed a lower level of commitment, while some primary care participants were less interested in the meeting’s contents:

“No, I think (that the seminar on chronic diseases) it did not favour the link between the levels (…) here I saw an attitude of some colleagues, kinda I already know that, I’ve been told that, come on! That it’s gonna be the same old rehash as usual” (Professional Platform/Secondary Care).

From the Local Steering Committee’s discourse, emerged that the joint evaluation (following each seminar) allowed adjustments in the content and dynamics, in order to improve involvement:

“Observing either participants or non-participants was going to give us a lot of feedback (teaching), while the satisfaction and the diagnostic surveys would let us know in which topics they were having the best results and so by exchanging our observations with the trainers – how you perceive this group, more proactive, less proactive – although we had that kind of feedback with them” (Local Steering Committee/Manager).

Likewise, being trained within the working hours, and the curricular value encouraged involvement (***Table 4e***):

“(Were required) the necessary time and adequate permission so that they could fully spend three days to be trained and also their labour times and rights were respected, you have to receive train during work hours” (Local Steering Committee/Manager).

**Healthcare network**. According to informants’ discourse, joint training meetings on maternal health, made possible by freeing up and protecting both planning and execution time, received the greatest institutional support. Physicians from both levels also received support to attend these joint training meetings (***Table 4f***). However, some informants also pointed out the limited support provided by some primary care and secondary care middle managers, who delayed physician assistance, due to work overload and shortage of personnel (***Table 4g***). Meanwhile, seminars on chronic diseases received the least support, and some were cancelled (***Table 4h***). At the individual level, the Local Steering Committee pointed out that less time working in the network encouraged primary care personnel to become involved and display a better attitude (***Table 4i***).

## Discussion

This study contributes to the knowledge on the implementation of participatory interventions to improve care coordination in health services by analysing the factors that influenced their implementation from the perspective of the participants and their effects on improving care coordination, which has been little explored internationally [21,22] and is non-existent in Mexico. Recently, Mexico has tried to move from vertical implementation towards a participatory approach, promoted by its health care policy that considers these types of interventions [7]. The results show the importance of the involment of professionals in the design of interventions, institutional support and methods reflexive training, as determinants to improve clinical coordination between levels.

Thus, a differentiated impact between interventions and topics is shown: joint training meetings on maternal health contributed to improve coordination of clinical management and of, as well as information interactional factors, and these training were replicated in other state healthcare networks (60 seminars, approximately 1600 participants). In contrast, joint training meetings on chronic diseases and the offline virtual consultations, in particular, did not greatly improved care coordination. These differences made it possible to identify process factors (adequate development of the participatory process in the design), context (institutional support and alignment with policies) and content (implementation of reflexive methods) that influenced the results yielded by the implemented interventions.

### Proper development of the participatory component is an essential element in adapting TO THE context

The results seem to suggest a limited involvement of professionals in the design and implementation process of the offline virtual consultations, which may have contributed to the selection of an intervention that did not meet the needs of participants, affecting its implementation and use. In the very early stages of the participatory processes, it is common to be oriented by the research team, and may result in decisions not resulting from consensus, decreasing the interest of professionals [15,16]. Furthermore, the barriers identified to implement the offline virtual consultations, such as complexity of the form and some physicians having limited ability to use computers, are coincident with those described in the adoption of technological tools by health professionals [43,44]. These barriers can be bridged if those who will use them are involved in the design [44].

Likewise, the importance of involving health professionals and managers when adapting interventions to a particular context was observed. Maternal health and the participation of managers resulted in an intervention that responded to institutional needs, and the participation of professionals had a positive impact on its adoption and effectiveness [20,21]. In contrast, for joint training meetings on chronic diseases, the lower involvement of managers may have affected their adaptation and limited their impact. The flexibility of the participatory action research method became relevant when it allowed the intervention to be adapted to incorporate another strategy for the new institutional objectives, which were aligned with the interest of professionals in the face of changing authorities.

### Institutional support is essential for development and results of interventions

It is well-known that managerial changes, derived from the political cycle or institutional modifications, influence healthcare priorities [25,45]. This was demonstrated in this study by the differentiated institutional support towards implementing the interventions and their results. The offline virtual consultations received little managerial support, which is essential for technological strategies, during its execution [44,46] due to the resources required. Meanwhile, as joint training meetings on chronic diseases were not an institutional priority, the low level of support received was evident, as just one seminar was conducted, while the rest of them were cancelled (this should also be considered when analysing their impact). This may have originated from the limited involvement of managers when designing these two strategies. On the other hand, the joint training meetings on maternal health were greatly promoted, as they were aligned with institutional interests and responded to a priority policy of the state [47]. This was coincident with other studies reporting that both alignment with priorities of the organisation and institutional support are determining factors for interventions to be effective, including participatory ones [10,48,49,50].

### Implementing Reflexive methods for joint training contributes to improvement in clinical coordination by influencing interactional factors

Participants noted improved trust and knowledge among primary care and secondary care physicians, according to the reflexive methods implemented in the joint training meetings, which are factors that influence care coordination by promoting the interest of professionals in collaborating and communicating with others levels of care [2,51]. Therefore, it is important to spread this type of intervention in order to improve clinical coordination by promoting agreement on treatments and establishing joint reference criteria, resulting in more appropriate and timely referrals [52,53], which occurred during the joint training meetings on maternal health, compared to conventional training [54,55,56,57]. This aspect is especially relevant for the Veracruz context, in which the results of the baseline study showed low values for mutual trust (11.5%) and personal knowledge (49.9%) among physicians in the healthcare network [3,4]. On the other hand, given that the offline virtual consultations were rarely used by primary care physicians, as they were wary of conducting interconsultations with unknown colleagues, the importance of promoting, before or in parallel, personal knowledge and trust between physicians from both levels of care, became evident. This aspect has been poorly explored in other studies on these types of interventions [44].

## Study limitations

The high turnover of health professionals, members of the Local Steering Committee and Professional Platform, caused that very few informants remained throughout the process of design and implementation of the interventions, so the opinion of some, regarding the factors that influenced and the results on coordination, could be limited to the moment and/or intervention in which they participated. These limitations were addressed by interviewing the participants from each phase; however, it could affect the depth of the information collected.

## Conclusions

The results show an uneven impact on cross-level clinical coordination of two participatory action research-based interventions, which were also different in design and implementation. Due to professional and managerial involvement in the design and flexibility of the participatory action research method, it was possible to adapt the interventions to the context of the healthcare network by aligning the needs of the professionals with institutional priorities in a complex scenario where needs were constantly changing. Likewise, the institutional support was a determining factor for their execution, development and results. Finally, implementing reflexive methods for training professionals in the health field contributed to strengthening interactional factors between primary and secondary care physicians, a key aspect to improve clinical coordination between levels of care.
